# Impact of vaccine mandate on Tdap vaccination coverage among Illinois students 2014-15

**DOI:** 10.11604/pamj.2017.27.103.10839

**Published:** 2017-06-09

**Authors:** Yaa Karikari, Oyinade Akinyede, Vanessa Davis, Kenneth Soyemi

**Affiliations:** 1 Department of Pediatrics, John H Stroger Hospital of Cook County, 1901 W Harrison Street, Chicago, IL 60612, United States

**Keywords:** Tdap vaccine, Illinois, exemption

## Abstract

In response to recent pertussis resurgence, a multi-agency recommendation that students receive a one-time Tdap vaccine was introduced. Post mandate there was sequential increase in the Tdap vaccine uptake in the targeted population.

## Brief

**Figure 1:**
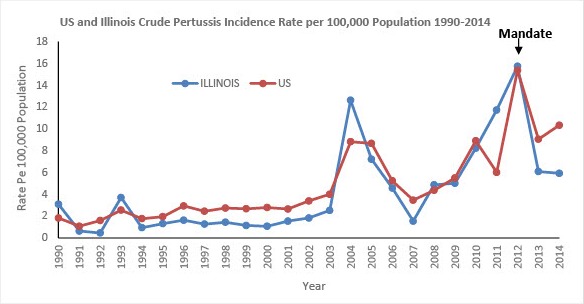
Graph showing US and Illinois pertussis rate per 100,000 population

Pertussis (whooping cough) is a highly contagious respiratory disease caused by the bacterium *Bordetella pertussis*. It was one of the common childhood diseases and a major cause of childhood mortality in the United States in the 20^th^ century before the introduction of the vaccine. The pertussis vaccine was first introduced in the 1940s initially as a whole-cell pertussis vaccine and became available combined with diphtheria and tetanus toxoids (as DTP) in 1948 [1]. The pertussis vaccine is now given with early childhood immunizations as the acellular pertussis vaccine in combination with tetanus and diphtheria toxoid as DTaP introduced in the 1990s [1]. A cellular pertussis-containing vaccines (Tdap) were first licensed for adolescents and adults in 2005 [1]. Protection however, from these vaccines begins to wane over time and puts pre-teens, teenagers, and adults at risk for the illness resulting in intermittent outbreaks. Since the introduction of the vaccine in the 1940s, with national case counts in the 100,000s per year, case counts declined to the 1000s in the 1970s and early 1980s [2]. The incidence of reported pertussis began increasing in the 1980s with significant peak in 2012 where 48,277 cases were reported nationwide, the highest since 1955 which recorded 62,786 cases [2] (Figure 1). Postulated reasons for resurgence and increase in pertussis incidence are multifactorial and some of the cited reasons are improved sensitivity of *Bordetella pertussis* real time PCR (rt-PCR) diagnostic testing, changes in the surveillance case definition (which improved sensitivity), increased provider and public awareness, mismatch of vaccine antigens and circulating strains, and reduced duration of immunity from the current acellular pertussis vaccines [3, 4]. In response to the aforementioned resurgence (accompanied by increase in school outbreaks and household clusters of infections) in 2012, the Illinois State Board of Health approved recommendations from the Illinois Department of Public Health (IDPH), the Illinois State Board of Education (ISBE) & the Immunization Advisory Committee to require all 6-12th grade students receive one Tdap vaccine [5] in concordance with the current practice in several states. ISBE classifies grades 6 through 12 students into the following three categories regarding DTP/DTaP/Td/Tdap vaccination status: 1) Protected and in compliance 2) Unprotected and in compliance and 3) Unprotected and noncompliant. Students who are found to be non-compliant are excluded from school until their health exams and immunizations are in compliance with the state laws. The McKinney Vento Act allows children and youths in Illinois opportunity to continue their education, without interruption, in their original school despite being displaced by homeless [6]. If parents or caregivers of these children and youths choose to enroll them in schools other than the school of origin, these children are enrolled immediately. Our study objective was to review post vaccine mandate Tdap vaccination coverage (uptake) in schools by comparing two data collecting systems (the ISBE and Centers for Diseases Control and Prevention (CDC) population based immunization survey data)); and to evaluate the reasons for non-vaccination in Illinois students during 2014-15 academic year. Aggregated school health examination and Tdap immunization compliance data (Grade 6-12) was extracted from ISBE website and the CDC population based immunization survey data from the CDC website. Qualitative variables were expressed as percentages. Quantitative variables were expressed as mean values (+/- standard deviation (SD)) if they followed a normal distribution or as median values (interquartile range (IQR)) if otherwise. Incidence Rate Ratios & the 95% confidence intervals (95% (CI)) for vaccine noncompliance exclusion were modelled using negative binomial regression.

**Figure 2:**
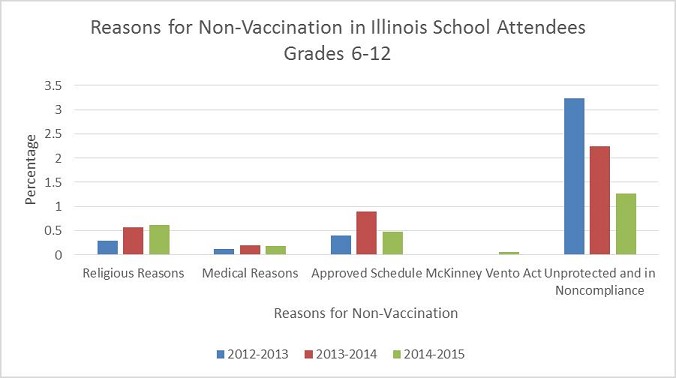
Graph showing the trend for vaccination exemptions over the last 3 school years

Aggregated data of 1,151,993 students was submitted from 3201 schools of which 2366 (74%) were public schools with a median (IQR) student enrollment of 211(64 - 395). Of the enrolled students, 1,121,953 (97.39%) presented documentation of obtaining Tdap vaccination. Religious exemption was obtained by 7,047 (0.61%), 5,362 (0.47%) presented a schedule from a physician or clinic indicating the date(s) to complete the required doses and 2,081(0.18%) presented a physician statement of immunity or medical objection. The McKinney Vento Act, which allowed homeless students to enroll in school while unable to submit completed records, was used by 925(0.08%). Students with no documentation of vaccination or exemptions were 30,040 (1.27%). Overall vaccination compliance was high with low Tdap exclusions among Illinois Grade 6-12 students. Negative binomial regression analysis showed higher Incidence Rate Ratios of vaccine noncompliance (3.7; 95% Confidence Interval (CI), 3.2-4.8) for public versus private school students. After the vaccine mandate, there was sequential increase in the annual Tdap vaccine uptake among persons’ age 13-17 years and Grade 6-12 students according to the Centers for Diseases Control and Prevention (CDC) population based immunization survey data and the ISBE data respectively. According to ISBE, vaccine coverage improved from 96.0% during the 2012-13 school year to 97.3% in the 2014-15 school compared with the CDC population survey’s vaccine coverage rates of 77.3%, 86.2%, and 91.9% for 2012 (pre-mandate), 2013, and 2014 respectively. Even though there were differences in slope increments, increases in vaccine coverage for both data systems were statistically significant using the chi square trend test (P < 0.01). Previous studies have demonstrated improved vaccine coverage after legislative mandate [7, 8]; in addition, improved vaccine coverage has been reported in the State of New Jersey with a pre-post vaccine mandate coverage spread of 47 percent points during a six-year interval. We are encouraged by the high vaccine uptake post mandate, as the accepted pertussis vaccination levels that will provide herd immunity is between 92 and 94 percent. However, we caution that high vaccination rates do not translate into reduced infection and outbreaks as recent studies of outbreaks among adolescents in California and Washington have confirmed that Tdap provides moderate protection against pertussis during the first year and then waned rapidly so that little protection remained 2-3 years after vaccination [9, 10]. The shifting epidemiology of pertussis, disease burden among adolescents, waning immunity and decrease vaccine effectiveness further away from vaccination means we can forecast future sporadic clusters and outbreaks among Illinois students. Health policy refers to decisions, plans, and actions that are undertaken to achieve specific health care goals within a society. An explicit health policy such as the state Tdap vaccine mandate was used to define a vision of improved regional and statewide Tdap vaccination uptake and rate which in turn will help establish targets and points of reference. It is important that school authorities and the medical community continue to work with families to encourage vaccine uptake, as the majority of exemptions in Illinois schools were due to religious (Figure 2); and that vaccine noncompliance was higher among public school students as compared with private schools. The reasons for higher noncompliance could not be ascertained because we had aggregated student data and could not analyze individual student’s reasons for noncompliance an important study limitation. To reduce noncompliance, expanding school physicals and vaccinations campaigns into schools will benefit students who cannot get a school exam (for reasons such as access to care or financial reasons) so they are not excluded from school as well as their parents who have problems scheduling and arranging appointments.

## Competing interests

The authors declare no competing interest.
